# A company’s relational strategy: Linkage between strategic choices, attributes, and outcomes

**DOI:** 10.1371/journal.pone.0254531

**Published:** 2021-07-22

**Authors:** Agnieszka Zakrzewska-Bielawska, Dagmara Lewicka

**Affiliations:** 1 Department of Management, Lodz University of Technology, Lodz, Poland; 2 Department of Business Management, AGH University of Science and Technology, Krakow, Poland; Univerza v Mariboru, SLOVENIA

## Abstract

Nowadays, the idea of firms’ atomization is rejected and companies are perceived as entities embedded in inter-organizational relationships and their configurations, including dyads and networks. The relational view in strategic management thus prompts research on a firm’s relational strategy. This paper taps this gap considering links between strategic choices and attributes of a company’s inter-organizational relationships, as well as the outcomes achieved by collaboration with different groups of stakeholders. We test the model based on research carried out on a representative sample of 400 enterprises operating in Poland and on international markets. The results of structural equation modeling show that 1) the outcomes of collaboration reflect market benefits and are dependent on the durability of the inter-organizational relationships and the heterogeneity of the supply chain relationships, 2) durability as an attribute of the relational strategy depends on the choice of how to create and appropriate value, and 3) in turn, the attribute of heterogeneity of the relational strategy depends on what type of partners are selected. Thereby, we deliver managerial implications on how to create a relational strategy to achieve a relational rent and better a company’s market position.

## 1. Introduction

“No business is an island” [1]. This famous statement by Scandinavian researchers underscores the fact that modern businesses operate as part of an ecosystem [2–6], whose participants are not doomed to adapt to their environment, but co-create it by establishing various types of inter-organizational relationships (IORs). Organizations have ceased to be atomic entities [7] and have become entities anchored in the systems of relationships (dyads and networks) [8], covering social, professional, and exchange relationships [9]. Therefore, competition for value from inter-organizational relationships [10–12] has become another pillar in the theory of strategy, and relational strategy was the basis for the company to achieve its benefits, including, above all, obtaining a relational rent [10, 13].

Taking the perspective of the relational view [14, 15] assuming that inter-organizational relationships as strategic resources are a source of competitive advantage, a relational strategy covers four basic strategic choices, such as 1) creating and appropriating value, 2) the choice of partners, 3) inter-organizational behavior manifested in cooperation and coopetition, and 4) the method of strategy creation [16]. These strategic choices are not made in isolation, but are closely connected to one another and interdependent, creating a portfolio of inter-organizational relationships that allows the company to achieve a relational rent [13, 17]. At the same time, these choices affect the main attributes of interfirm collaboration.

Inter-organizational relationships are diverse and can be classified according to different criteria [18]. Initially, they were classified according to exchange [19], commitment [20], and reciprocity [21] which should be considered together. Researchers have also analyzed them, both because of the relationships *per se* (e.g., reality, continuity, directness, interdependence, symmetry, permanence, benefit, and formality) and because of the criteria that apply to the participants of the relationship (e.g., position in the sector, position in the value chain, inter-organizational behavior of the companies, or complexity, coordination, and asymmetry of the power of the participants; see, for example [22–28]). This wide variety of relationships between organizations raises the question of which of their characteristics are so crucial that they can act as the attributes of a relational strategy. Assuming the transitivity of the attributes of both inter-organizational relationships and relational strategies, which expresses the conviction that relational strategies are distinct from the strategies formulated so far [29] and on the basis of expert research, we have assumed that these attributes include durability, heterogeneity, originality, and formalization of IORs. It should be noted that the expert study was conducted in 2018. Twenty experts, representatives of the Polish scientific community, were invited to take part in the research. They were selected based on the quantity and quality of their scholarly work in the area of strategic management and/or issues related to inter-organizational relationships or networks. The experts were asked about what attributes of individual relationships should represent and describe the strategic attributes of interfirm collaboration as a whole. The results of this study indicated four key, strategic attributes of interfirm collaboration [30].

Inter-organizational collaboration leads to specific outcomes. The research to date has focused primarily on identifying its benefits, indicating that cooperation and coopetition contribute to achieving synergy, gaining access to resources, sharing resources, or generally improving the performance of an organization through collaboration (e.g. [31–34]). Meanwhile, inter-organizational collaboration may also result in a loss of potential opportunities or destruction of value [35]. Therefore, it may involve risks, costs, and negative consequences [7, 36–38].

The linkage between the strategic choices, attributes, and the outcomes achieved through interfirm collaboration has so far not been the subject of deeper scientific explorations in the widely discussed topic of business networking, thus there is a research gap. The paper aims to fill this gap by realizing four main goals. The first one is recognizing a company’s strategic choices as perceived by top management. The second one is assessing the impact of those choices on the particular attributes of the company’s relational strategy. The third one is diagnosing the outcomes achieved by a focal company from inter-organizational collaboration. The last, the fourth goal is to explore the relationship between a company’s strategic choices, attributes of relational strategy, and outcomes of interfirm collaboration. It should be noted that each strategic choice made as part of a relational strategy may have a greater or lesser impact on its individual attributes. In our research, however, we assumed that the individual attributes of a relational strategy are linked to the specific strategic choices that have the greatest impact on them, and they were further analyzed in this context. We used survey data gathered from 400 companies based in Poland and operating either in Poland or internationally. The sample was representative for Poland in terms of company size, and we used multi-mode method for data collection.

We contribute to the literature on strategic management from the perspective of the relational view by 1) recognizing how managers perceive strategic choices of a firm’s relational strategy, 2) identifying the attributes which characterize a firm’s relational strategy, 3) diagnosing the outcomes (positive and negative) from interfirm collaboration, and consequently, (4) developing a comprehensive framework that covers the linkage between the strategic choices, attributes, and outcomes of firms’ relational strategies. These contributions are helpful for uncovering different associations by which managers can successfully build a strategic inter-organizational collaboration to achieve a competitive advantage and relational rent.

In the first part of the study, we explain the theoretical basis of the linkage between the strategic choices, attributes, and outcomes of a relational strategy, formulate hypotheses, and build a conceptual model. Then, we present the research methodology and the results of our analyses. In the final part, we discuss and point out the main research limitations and, based on them, propose directions for further research.

## 2. Theoretical background and hypotheses

### 2.1. Value creation and appropriation and durability as the attribute of the relational strategy

The relational/network context of contemporary enterprises has triggered the need for research into the creation and appropriation of relationship value, becoming one of the main problems of strategic management [39, 40]. Value creation is defined as the total sum of value that is generated collectively through the joint effort of different partners (market stakeholders), while value appropriation is defined as the individual share of the value that a firm can capture [41]. From the perspective of strategic choices, companies can create value according to the logic of the value chain [42, 43] or the logic of the value network [34, 44]. The former refers to the concept of the economic path and means that every company occupies a position in the chain; upstream suppliers provide inputs before passing them downstream to the next link in the chain, their customer [44]. Value in this case is created on the basis of vertical/sequential interdependence: by each actor one after another [45]. In turn, value network logic focuses on creating value together [46]. A focal company and its different economic partners (suppliers, customers, competitors, or complementors) work together to co-produce value, which benefits each of them [47].

Regarding appropriating value, strategists must decide by which mechanism the value will be captured. Previous research in this regard provides many solutions that have been identified and analyzed from the perspective of various theories and streams, e.g., industrial marketing, resource-based view, game theory, stakeholder theory, justice theory, or negotiation theory [48, 49]. Generally, a focal firm may choose between mechanisms for protecting value and mechanisms for maximizing value. For value protection, isolating mechanisms are most commonly used [50]. These include both formal mechanisms—such as patents, copyrights and intellectual property rights, and formal contracts—and informal mechanisms such as confidentiality, secrecy, or hidden knowledge [51–53]. The value protected in this way does not increase the economic rent, but it rather aims to limit losses. Therefore, instead of protecting generated value, appropriation can be accomplished by maximizing value. For this purpose, the mechanisms for reconfiguring the resource base by efficiently acquiring, integrating, and releasing the resource bundle to obtain a relational rent are the ones most often mentioned [10, 17].

Value creation and appropriation as a result of collaboration are closely related and represent “two sides of the same coin.” The value created in a collaborative effort among exchange partners is further appropriated, wherein a focal firm might achieve superior value because of superior value creation, superior appropriation of the value pie, or a combination thereof [54]. The way value is created and appropriated affects the durability of the relational strategy, expressed in purposeful development, maintenance, or withdrawing from inter-organizational relationships [22]. The relational strategy maintains long-term and permanent relationships which will yield a relational rent for the company but eliminates those relationships which cease to be a source of relational rents. However, this requires long-term effort towards the relationships, trust [55, 56], commitment in cooperation [57, 58], or overcoming difficulties in maintaining relationships [59]. Value created according to the logic of the value chain assumes chain interdependence, in which entities focus primarily on logistical synchronization by solving exchange problems and unilateral learning when anticipating them [43, 60, 61]. In essence, the relationships established and maintained are of a transactional nature [62] focusing on getting the most out of individual transactions. However, as they are repeated, they may take on a forward-looking character, i.e., they may present potential for development towards long-term relationships. In the case of value network, a company and its partners are required to cooperate and constantly adapt to changing external and internal exchange conditions [63]. The collaborating entities are interdependent, and the quality and durability of their relationship depends on the commitment of the different entities and mutual trust. This impacts the effectiveness of the exchange and the satisfaction of the partners, while determining the durability of the relational strategy through decisions to maintain or break up the relationship.

On the other hand, the durability of inter-organizational relationships, and therefore of the relational strategy, depends on the direct and intentional efforts of the parties involved at capturing value [48] and the proportions and principles of its appropriation [64]. The mechanisms of protecting the values are primarily geared towards reducing losses [65]. They protect the company’s valuable resources, especially knowledge, but they also generate high costs. They do not foster an increase in economic rent, thus limiting the durability of IORs, and further, that of the relational strategy. On the other hand, the mechanisms of value maximization cause an increase in the economic rent through inter-organizational synergy and affect trust and satisfaction with the relationships, which may result in it being more durable.

In light of the above, having analyzed the durability of collaboration in the context of choosing the logic of value creation and the mechanisms of its appropriation, we put forward the following hypotheses:

H1a: The value created in the value network improves the durability of the relational strategy
H1b: The value appropriated by maximizing mechanisms improves the durability of the relational strategy.

### 2.2. Choice of partners and heterogeneity as an attribute of relational strategy

The second strategic choice in shaping a company’s relational strategy is the selection of key partners. The above selection involves two thought constructs: the type of partners in a relationship and the criteria for selecting these partners. The type of partner is usually defined from the perspective of stakeholders, understood as groups or individuals that can influence the achievement of the organization’s goals [66]. In the case of relational strategy, market (external) stakeholders—i.e., those operating in the company’s environment—are analyzed [67]. External stakeholders are subject to different classifications. For example, Scuotto et al. [68] propose a division of partners into two categories: market partners and scientific partners. The former include customers, suppliers, and competitors, while the latter include universities and research centers. In turn, Sofka and Grimpe [69] and Ritala et al. [70] identify market partners (customers and competitors) and technology partners (universities, research centers, and consultants). However, the most common criterion used to classify types of relational partners is the economic path. Based on this criterion we can distinguish two categories of partners: partners on the sector’s economic path and partners outside the economic path [71, 72]. The first category includes suppliers, customers, and competitors; the second category includes all entities outside the economic path, e.g., research institutes, companies from other sectors, social, financial, or governmental organizations, etc. Despite the lack of a single, universal typology of partners that would be used by all researchers, certain types of relationship partners are universal in nature, as they are included in most classifications, regardless of the division criterion adopted. These include suppliers, customers, and competitors, the latter only being stakeholders when there is an exchange relationship with the company [73].

The second construct considered in the framework of selecting key collaboration partners is the criteria for partner selection. As with definitions for stakeholders, there is no consensus on the set of criteria that companies should apply in this selection. Researchers have indicated strategic and technological alignment [74], resource alignment [75, 76], competitive position and common goals [77], personal preferences and personal compatibility [78], trust [56, 79], cultural similarity [80], reputation [81], financial standing, cost, and quality [82], and many more. Most researchers adapt the list of criteria for partner selection to the type of collaboration discussed in a given study, or take into account the selected criteria to emphasize their particular role. Furthermore, Pidduck [75] stresses that choosing a partner is a complex process, often occurring through complex negotiations rather than a rigid and rational procedure, which is also determined by the company’s previous experience.

The selection of key partners by their type and selection criteria influences the attribute of heterogeneity of a relational strategy, expressed by the complexity and multiplicity of inter-organizational relationships [83, 84]. Maximizing the number of relationships—with different partners and with different objectives and resource profiles—helps to gain an advantage using a relational strategy, though the existence of many different relationships requires consideration of their interdependence [1]. Taking into account the strategic selection of key partners in the context of the heterogeneity of the relational strategy, we have formulated the following hypotheses:

H2a: The more actively a company develops its inter-organizational relationships with different groups of partners, the more heterogeneous the relational strategy.
H2b: The more varied the criteria for partners’ selection, the more heterogeneous the relational strategy.

### 2.3. Inter-organizational behavior and originality as an attribute of relational strategy

Inter-organizational relationships can take one of four forms: coexistence, competition, cooperation, and coopetition [24]. Of these four types, only two (cooperation and coopetition) are based on collaboration, including the convergence of interests, and are a source of relational rents. Cooperation is defined as a relationship in which individuals, groups, and organizations interact by sharing complementary capabilities and resources, or leverage these for the purpose of mutual benefit [85]. It is a kind of collaboration with non-competitive partners (suppliers, customers, and complementors). Cooperation can be considered from the transactional or relational [51], which is also known as market-focused strategy and relationship-focused strategy of cooperation [86, 87]. From the transactional view, cooperation relies on formal rules and objectives [88]. The basis of a transactional relationship is the transfer of products of a certain value [89], and a continuing relationship requires the use of various types of price incentives, which encourage repeat transactions and determine its future value [90]. The effectiveness of incentives depends on the contractual provisions and—as Malhotra and Lumineau [91] point out—contracts based on extensive and detailed regulations and aimed at controlling and minimizing the risk of opportunistic behavior reduce the goodwill within the relationship and the willingness to continue the relationship [57]. From the relational view, cooperation is strongly determined by repeated interactions and exchanges between the partners that establish a mutual understanding, social identification, and trust between them [88]. This kind of cooperation is long-term and includes non-competitive partners with whom a firm has developed trust through existing ties; e.g., friends, acquaintances, and their recommendations [87]. Trust-based cooperation (partnership) improves communication and the exchange of information between partners [92] and creates an atmosphere of mutual understanding for the contract to be carried out within, which is particularly important in unpredictable situations [19].

On the other hand, collaboration with competitive partners that simultaneously combines cooperation and competition between enterprises is called coopetition [93]. Scholars have indicated many different drivers, both external and internal, which lead to coopetition [94]. Generally, coopetition is formed and developed either by the will of firms or on demand (through pressure). Voluntary coopetition is often driven by high uncertainty, shorter product lifecycles, the convergence of multiple technologies, and increasing R&D expenditures [95, 96], as well as access to resources, expanding markets, and the desire to improve efficiency or competitive position [97]. Coopetition is also a result of institutional, competitive, and customer pressures [94]. The influence of customers on the formation of coopetition is particularly important, and buyers often explicitly demand that competitors cooperate in order to get the best possible product, by combining technologies of the respective coopetitors.

Cooperation and coopetition are elements of the value network and sources of relational rent. The distinguishing feature of relational rent is idiosyncrasy, which means that the rent is closely linked to a specific relationships or specific partners. In this sense, the relational rent is non-imitable and rare in its originality [30]. Originality as an attribute of a relational strategy is the result of selecting exceptional partners (both competitive and non-competitive) and configuring the relationships established. Different configurations of relationships may arise around the company, such as chain or network relationships, which are deliberately shaped to achieve strategic goals [98]. Inter-organizational relationships may lead to the creation of relatively open structures such as a network, but also to much more enclosed ones because the social closeness (clique), or because of familial bonds (clans) [99]. Therefore, the originality of the relational strategy is fostered by the relationship arrangements covering both cooperation and coopetition relationships; however, due to social embeddedness [100] and long-term intent, these are primarily the relationships of partnership and voluntary coopetition. Therefore, we formulated another hypotheses:

H3a: The more inter-organizational relationships are based on partnership cooperation, the more original the relational strategy is.
H3b: The more inter-organizational relationships are based on voluntary coopetition, the more original the relational strategy is.

### 2.4. A way of establishing relational strategy and formalization as its attribute

The last strategic choice is the method of establishing the relational strategy. The way a strategy is created is one of the most disputed issues in strategic management [101]. The importance of strategic analysis [102] supports the view that strategy is a result of rational/analytical processes directed by top managers. Such strategies are referred to as deliberate strategies because they arise from deliberate decisions [103]. On the other hand, strategies may emerge through adaptation to circumstances [104], as indicated by evolutionary theory, thus being the result of ad hoc, incremental, or even accidental actions taken at the lower levels of management. The process of establishing them is an effect of experimentation and searching for the best way to develop, and those strategies are called emergent. However, in practice, the process of creating a strategy is almost always a combination of centrally created rational design and decentralized adaptation [105]. Similarly, the relational strategy combines these two approaches. On the one hand, inter-organizational relationships and their systems are formed intentionally (they are a conscious choice of decision-makers indicating whether to establish, maintain, develop, or withdraw from a given relationship), while on the other hand, they are emergent in nature and are shaped from ad hoc actions and opportunities that arise.

Inter-organizational relationships can be divided into formal (contract-based) and informal (trust-based) [106]. Managers make deliberate choices between formal and informal relationships, and then agree on this choice with each partner. Therefore, the degree to which the relationships are formalized—distinguishing between contractual relationships and social relationships—appears to be another attribute of the relational strategy [107]. It should be noted that formal relationships are an attempt to design the future and a way of responding to uncertainty, by reducing the ambiguity of relationships. Thus, we assume that formal relationships are characterized by a relational strategy shaped by a predominantly deliberate approach, while informal ones based on trust and social ties more often result from a mainly emergent approach. Therefore, we formulated another hypothesis:

H4 – The more the relational strategy is deliberately shaped, the more it is characterized by formal relationships.

### 2.5. Company outcomes from collaboration

The desire to achieve a relational rent is the foundation for creating a relational strategy for a company. The question, however, arises here as to what effects (viewed from the point of view of benefits and costs) its implementation will bring. The outcomes of a collaboration may be tangible or intangible [108], they can constitute a mutual benefit or benefit only one firm [109], and they then can contribute to both the focal firm’s performance or the performance of the alliance/network [15]. The research to date has focused primarily on identifying the benefits of inter-organizational collaboration, indicating that from the focal firm’s perspective, cooperation and coopetition contribute to achieving, in particular, 1) resource outcomes, including access to partners’ various resources—especially knowledge and sharing and creating shared resources (see, for example [110, 111]); 2) financial outcomes, including reduced operating cost—especially transactional costs, increased revenues, or the option of acquiring funds for shared investment (e.g. [112–114]); 3) organizational outcomes, which include primarily improvements to processes such as better quality, faster delivery time, or greater flexibility [115] and more innovation [116]; and 4) positional outcomes, including greater bargaining power of the entities compared to those outside the inter-organizational system/network [65, 117].

Meanwhile, the implementation of a relational strategy may involve risk, costs, and consequences as well as benefits (e.g. [7, 37]). Similarly, in this case they can be classified into four groups [118]: 1) dependency outcomes, which manifest themselves in a dependence on partners in the relationship, the imposition of collaboration conditions by the stronger entities, and the “blurring” of responsibilities (e.g. [119, 120]); 2) financial outcomes, which are the inverse of the benefits achieved, and therefore are expressed in increased operating costs or reduced revenues [36]; 3) organizational effects, which may have the opposite effect to benefits, i.e., a worsening of the processes involved or less flexible operation [121]; and 4) positional effects, which express a reduction in the bargaining power compared to the entities outside the inter-organizational arrangement [65]. In addition, the frequently indicated risks of cooperation and/or coopetition include a risk of knowledge leakage [106], conflicts between partners [122], and the possibility of opportunistic behaviors appearing [123, 124].

Which outcomes a focal firm achieves from organizational collaboration depends on many factors determining this collaboration, including strategic choices that affect the attributes of the relational strategy. We assume that long-term interfirm relationships with diverse partners, based on trust and partnership, taking into account difficult-to-imitate configurations, often shaped in an emergent way, should favor the benefits of collaboration and reduce the associated risks and consequences. Therefore, we formulated further two hypotheses:

H5 – The more a relational strategy is characterized by a) durability, b) heterogeneity, and c) originality, and the less by d) formalization, the stronger the positive outcomes a focal firm achieves from interfirm collaboration will be.
H6 – The more a relational strategy is characterized by a) durability, b) heterogeneity, and c) originality, and the less by d) formalization, the less the negative outcomes a focal firm achieves from interfirm collaboration will be.

**Fig 1:**
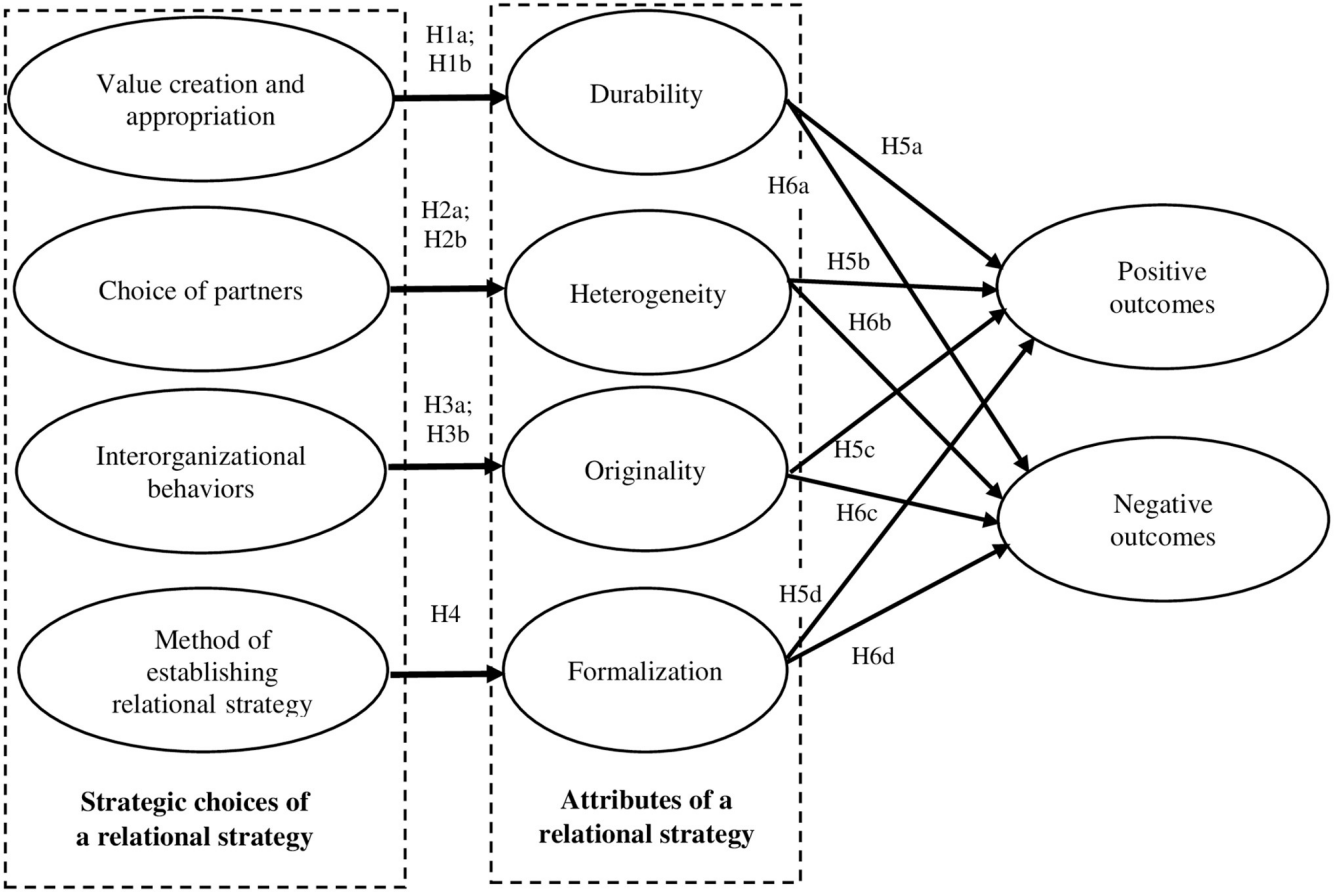
Conceptual model.

Putting together the strategic choices and the attributes of the focal firm’s relational strategy with the outcomes achieved from interfirm collaboration, we built a conceptual model presented on Fig 1.

## 3. Methodology

### 3.1. Sample and data collection

The research was conducted from December 2018 to May 2019. The study population included small (with 10–49 employees), medium-sized (with 50–249 employees), and large (250 or more employees) companies. Micro-companies (with up to 9 employees) were deliberately eliminated, as such entities typically have fewer and less complex inter-organizational relationships due to their limited resources and scope of operations. In 2018 in Poland, the total number of companies—excluding micro-companies—there were 52,662 small entities (73.6%), 15,210 medium-sized entities (21.3%), and 3,674 large companies (5.1%) [125]. Our goal was to construct a sample statistically representative. Therefore, we used a probabilistic, random-layer selection to ensure representativeness of the sample and to generalize the results [126]. We also used available sample size calculators, assuming a confidence level of 95% and a margin of error of 5% [127]. The results showed that the minimum required sample size was 382 entities in the case of the first calculator [128], and 383 entities in the case of the second calculator [129]. The survey frame was the National Economy Register (REGON) database, from which a gross sample was taken, i.e., a list of entities several times larger than the assumed sample population (the initial database consisted of 1,856 records). We obtained a total of 400 complete survey responses. In this regard, the sample was representative for Poland in terms of company size. The response rate was 21.5%, which is comparable to other inter-organizational studies, with response rates ranging from 10% to 30% (e.g. [88, 130]).

Additionally, the sector (production, retail, or services) and industry were taken into account according to the Polish Classification of Activities [131] in order to eliminate excessive concentration in one area, e.g., manufacturers in a given industry.

The research was conducted using the multi-mode method, which combines Computer-Assisted Telephone Interviews (CATI) and Computer-Assisted Web Interviews (CAWI); this translates into higher-quality data, in terms of precision, accuracy, error rate, and reliability [132]. The research tool was a standardized questionnaire, and the respondents were business owners (65.5%) or representatives of top management (33.5%). We received verbal consent from respondents for participating in our research and this consent was informed. Table 1 presents a profile of the surveyed companies.

**Table 1:** Profile of responding companies.

Size of firm:	N	%	Age of firm:	N	%
small companies medium-sized companies large companies	331 56 13	82.75 14.0 3.25	up to 5 years 6–10 years 11–20 years over 20 years	26 59 181 134	6.5 14.75 45.25 33.5
Dominant type of activity:	N	%	Market:	N	%
production services retail	194 105 101	48.5 26.25 25.25	domestic international	269 131	67.25 32.75

Note. N = 400

Most of the studied firms were mature entities, i.e., ones that have been operating on the market from 11 to 20 years (45.25%) or for over 20 years (33.5%), which means that they have experience in forming, developing, or withdrawing from inter-organizational relationships. In almost half of the firms, activities related to production prevailed, and the majority of the firms were active on the domestic market (67.25%).

To test for nonresponse bias, we examined differences between early and late respondents [133] on key company characteristics such as the age and size of the company and the dominant type of activity (divided into production, services, and retail). We took into account the first and last 25% of the respondents in our sample. The results of the U Mann–Whitney tests for company age and size, and a chi-squared test for dominant type of activity did not reveal any significant differences at a 5% significance level. Thus, our results should not be affected by a nonresponse bias.

We also checked the common method bias to avoid any issues for self-reported data from a single informant. Following the procedures recommended by Podsakoff et al. [134], we took several approaches, including 1) using multiple-item constructs to capture all of the key variables and grouping the construct items in sections instead of variables, 2) assuring respondents that there were no right or wrong answers and encouraging them to respond as honestly as possible, 3) avoiding ambiguous questions and vague concepts and keeping questions as simple as possible [135]. Moreover, we used the following methods: Harman’s one-factor test, confirmatory factor analysis (CFA), and validation of the model’s fit to examine the severity of common method bias as Podsakoff et al. [134] suggested. Our analysis, i.e. an unrotated principal component factor analysis, principal component analysis with varimax rotation, and principal axis analysis with varimax rotation as well as CFA results showed that the single-factor model did not fit the data well. Thus, there is little threat of common method bias in our study.

### 3.2. Measures

We prepared the survey items based on the literature. Simplicity in scoring was achieved using a 7-point Likert scale ranging from “strongly disagree” to “strongly agree.” We performed both exploratory and confirmatory factor analyses to validate the measures of the study. We conducted an exploratory factor analysis (EFA) to reduce the items down to resulting variables. We then conducted a CFA to assess the unidimensionality of each construct. We employed a rigorous process to purify and validate the measurement scale items, as advocated by Gerbing and Anderson [136] and Hair et al. [137], assessing the validity and reliability of particular constructs.

#### 3.2.1 Value creation and appropriation

To measure value creation, we proposed six items related to value chain or value network logic in the context of benefits achieved [45]. Value appropriation was measured by nine items describing both the mechanism of the protecting value [51, 52] and the mechanism of the maximizing value from a resource-based view [17]. Based on the EFA results, we reduced two items; next, we conducted a CFA that resulted in the extraction of four factors, which we named value creation by value chain logic (VCC), value creation by value network logic (VCN), value appropriation by value protection (VAP), and value appropriation by value maximization (VAM).

#### 3.2.2 Choice of partners

The choice of partners refers to the type of partners and the criteria for their selection. Based on previous research [73, 76, 81], we proposed ten items measuring the choice of partners. Following the EFA results, we discarded two items; following the CFA analysis, we extracted two factors, one relating to partner type (PT), and the second relating to criteria of partner selection (CPS).

#### 3.2.3 Inter-organizational behavior

This construct was described by 12 items related to the nature of the cooperation with suppliers, customers, and other entities [88], as well as coopetition [94, 96]. We discarded three items based on the EFA results, then we conducted a CFA, which extracted three factors: transactional cooperation (TC), partnership cooperation (PC), and coopetition (Coo). In the latter case, the items describing the coopetition form a single factor, taking into account both voluntary coopetition and coopetition requested by customers.

#### 3.2.4 Method of establishing a relational strategy

Here, we used six items related to the way a strategy is created: three describing the deliberate way [103] and three for the emergent way [104]. Two items were discarded as a result of the EFA. We conducted a CFA and obtained two factors, consisting of two items each. The first was the deliberate method of relational strategy creation (DW), and the second was the emergent way (EW).

#### 3.2.5 Durability

We measured durability as an attribute of the relational strategy by five items related to the duration of relationships, trust and commitment of partners, and the benefits that a focal firm achieved or may achieve in the future [30]. We reduced one item based on the EFA, and extracted one factor thanks to the CFA results.

#### 3.2.6 Heterogeneity

Based on the literature, we proposed seven items related to the complexity and multiplicity of inter-organizational relationships [83, 84]. The EFA resulted in the extraction of two factors in this case, and one item was removed. This was confirmed by the CFA. We named the first factor supply chain relationships (SCR) and we named the second one value network relationships (VNR).

#### 3.2.7 Originality

To measure originality as an attribute of the relational strategy, we proposed three items defining the forms and principles of difficult-to-imitate collaboration [99]. As a result of the EFA, we discarded one item and the remaining two were extracted in one factor, confirmed by the CFA.

#### 3.2.8 Formalization

We used three items related to formalization as an attribute of the relational strategy [107]. The EFA did not extract any factors here. Therefore, we decided to measure formalization by a chosen item (i.e., our strategy is based primarily on formal relationships as contracts) as an observable variable.

#### 3.2.9 Outcomes

We proposed ten items for positive outcomes and ten items for negative outcomes of collaboration related to resource, financial, organizational, and positional benefits or risks/costs [36, 65, 111]. In the case of positive outcomes (PO) of collaboration, the EFA extracted one factor and three items were discarded. This factor mainly took into account market, image, and position benefits. In the case of negative outcomes of collaboration, the EFA did not extract any factors, which is due to the fact that the respondents indicated their existence in isolated cases and all answers were therefore comparable.

We tested the convergent and discriminant validity of the study scales. Convergent validity was assessed by standardized path loadings and the average variance extracted (AVE). The loading factors of all items within each scale exceeded 0.5 and were statistically significant [137]; the AVE values were higher than 0.5 [136]. Similarly, the discriminant validity of the key constructs was satisfactory, since the square roots of the AVE values were greater than all individual correlations [137, 138]. The reliability analysis was conducted by calculating Cronbach’s α and composite reliability (CR) for each scale. The result shows that Cronbach’s α and CR for all scales surpassed the threshold value of 0.6 [138], suggesting good reliability.

Our variables with measurement items, factor loadings, convergent validity, and reliability assessment are presented in the S1 Appendix.

### 3.3 Data analysis

The hypotheses were tested by structural equation modelling (SEM), which offers the advantage of flexibility in matching the theoretical model with the data and allows the researcher to describe unobservable latent variables [139]. In the case of formalization as an attribute of a relational strategy, it was impossible to define a latent variable. That is why we used the observable variable here. To conduct various statistical tests, we employed two statistical packages: Statistica and Amos.

## 4. Results

The descriptive statistics and correlations for the strategic choices of the relational strategy, its attributes, and positive outcomes are reported in Table 2. Most correlations were significant and positive.

**Table 2:** Descriptive statistics and correlations between variables.

a) Strategic choices of relational strategy												
*No*.	*Variables*	*1*	*2*	*3*	*4*	*5*	*6*	*7*	*8*	*9*	*10*	*11*
1	VCC	**0.878**										
2	VCN	0.787**	**0.928**									
3	VAP	0.183**	0.161**	**0.883**								
4	VAM	0.471**	0.490**	0.207**	**0.832**							
5	PT	0.828**	0.784**	0.190**	0.495**	**0.829**						
6	CPS	0.348**	0.350**	0.101*	0.220**	0.382**	**0.860**					
7	TC	-0.247**	-0.254**	-0.055	-0.193**	-0.241**	-0.226**	**0.743**				
8	PC	0.305**	0.321**	0.101*	0.301**	0.277**	0.260**	-0.695**	**0.753**			
9	Coo	0.119*	0.101*	0.094	0.039	0.092	-0.010	-0.150**	0.187**	**0.724**		
10	DW	0.354**	0.342**	0.061	0.214**	0.394**	0.171**	-0.060	0.100*	-0.088	**0.828**	
11	EW	0.328**	0.317**	0.052	0.163**	0.271**	0.138**	-0.205**	0.205**	0.160**	-0.533**	**0.895**
	Mean	5.208	5.140	3.439	4.419	5.277	5.796	4.733	3.475	2.756	3.695	3.700
	s.d.	1,129	1.212	1.620	1.324	1.231	0.629	1.137	1.145	0.851	1.219	1.479

b) Attributes and positive outcomes of relational strategy							
*No*.	*Variables*	*1*	*2*	*3*	*4*	*5*	*6*
1	D	**0.888**					
2	SCR	0.758**	**0.894**				
3	VNR	0.214**	0.326**	**0.807**			
4	O	0.672**	0.501**	0.142**	**0.789**		
5	F	0.138**	0.215**	0.193**	0.088	-	
6	PO	0.507**	0.554**	0.269**	0.304**	0.205**	**0.720**
	Mean	5.066	4.923	3.173	4.614	3.373	4.518
	s.d.	1.141	1.004	1.046	1.411	1.369	0.778

Note. N = 400; s.d.–standard deviation; correlation is statistically significant at *p* <0.01 (**) or *p*<0.05 (*); the diagonal values (in bold) present the square roots of AVE

Note. N = 400; s.d.–standard deviation;

**correlation is statistically significant for *p* <0.01; the diagonal values (in bold) present the square roots of AVE

**Fig 2:**
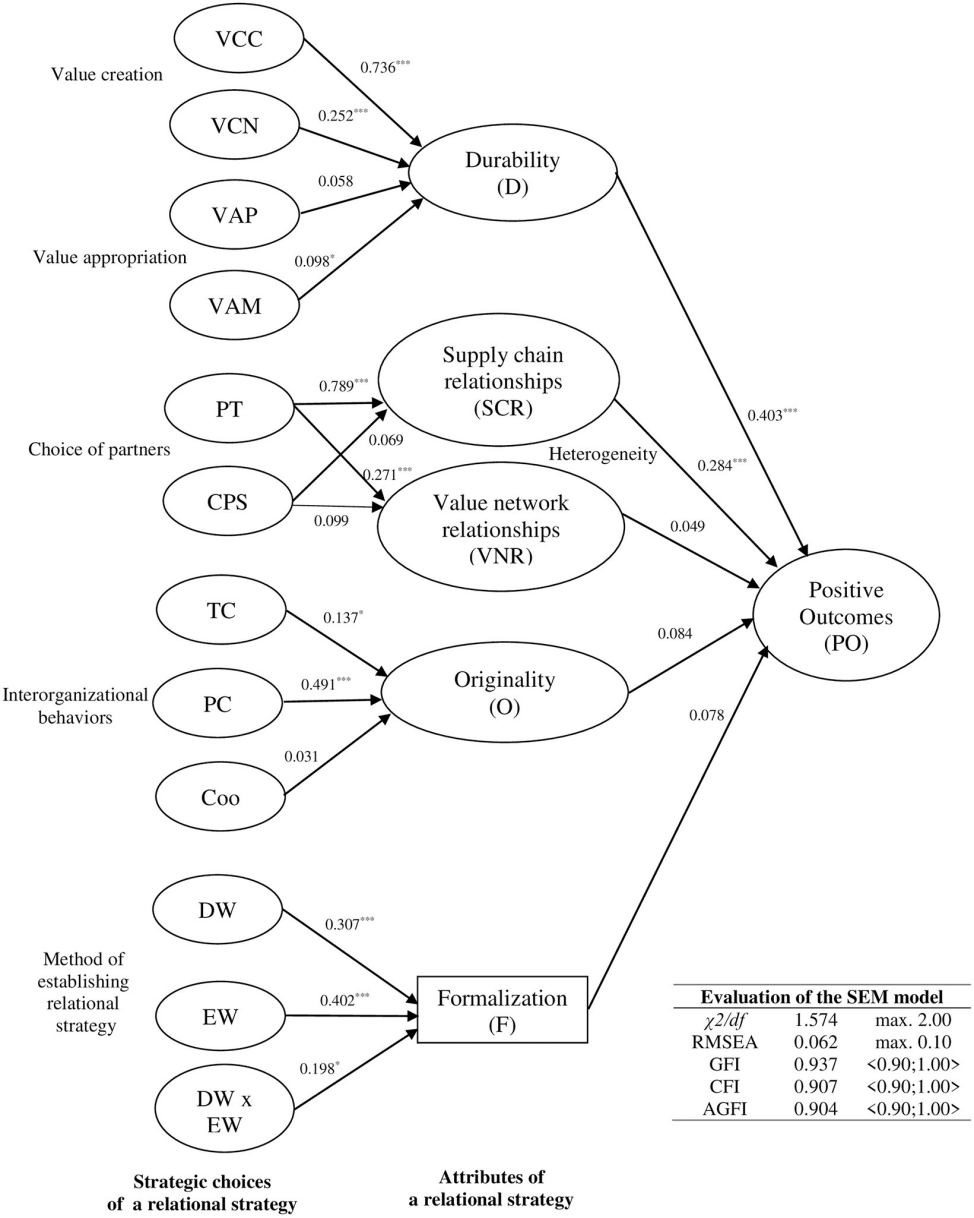
SEM model. Note: ***p<0.001; ** p<0.01; * p<0.05.

To test our hypotheses, we used SEM with maximum likelihood (ML) estimation and covariance matrix as data input. The ML estimation method is often indicated as well suited to theory testing and development [136]. SEM model for the links between strategic choices, attributes of the relational strategy, and outcomes achieved from the collaboration is presented in Fig 2. The model fit statistics were satisfactory.

As shown in Fig 2, durability (D) as an attribute of the relational strategy is significantly associated with value creation by value network logic (VCN) (H1a: γ = 0.252; t = 6.7; p<0.001) as well as with value appropriation by value maximization (VAM) (H1b: γ = 0.098; t = 2.937; p<0.05) that confirms the hypothesis H1. However, there is also another dependency between value creation by value chain logic (VCC) and the durability of the relational strategy (γ = 0.736; t = 13.03; p<0.001). Our results show that both ways of value creation impact on durability of relational strategy, but the impact of value creation by value chain logic is much stronger than the impact of value creation by value network logic.

Hypothesis H2 was partially confirmed. The type of partners (PT) is significantly associated with the heterogeneity of the relational strategy, as expressed by supply chain relationships (SCR) (H2a: γ = 0.789; t = 15.390; p<0.001) and by value network relationships (VNR) (H2a’: γ = 0.271; t = 3.481; p<0.001). The criteria of partner selection were insignificant, so we were unable to confirm Hypothesis H2b.

Hypothesis H3 was also only partially confirmed. Partnership cooperation (PC) based on long-term relationships significantly correlated with the relational strategy’s originality (O) (H3a: γ = 0.491; t = 7.928; p<0.001). Surprisingly, the originality is also influenced by transactional cooperation (TC) based solely on price (γ = 0.137; t = 3.876; p<0.05), which can be explained by the ability to capture price opportunities that are unavailable to others. Coopetition does not impact on this originality, probably because the companies in question rarely established coopetitive relationships.

Hypothesis 4 was falsified. Formalization (F) of the relational strategy significantly correlated with both the deliberate (DW) and emergent (EW) ways of strategy creation, and with the combination of the two (DW x EW). This shows that regardless of whether inter-organizational relationships are formed deliberately and according to a plan, spontaneously and in reaction to conditions, or through a combination of the two [101], most of these relationships are contractual and formally established [106]. This confirms the distrust among Polish managers towards collaboration partners who prefer formal relationships to social ties. It should be noted that distrust always accompanies trust [140, 141]. It is not a dangerous and destructive element of inter-organizational relationships, but a natural state characteristic of perceptive and prudent partners, who are aware of the mechanisms that direct people and their activities [142]. In this context, formal relationships protect partners from opportunistic behavior aimed at benefiting oneself at the expense of others [143].

Hypothesis 5 was partially confirmed (Fig 2). Only two factors significantly correlated with the positive outcomes of collaboration. Durability (D) as an attribute of the relational strategy influences the benefits achieved from collaboration (H5a: γ = 0.403; t = 7.665; p<0.001) as does the heterogeneity of the relational strategy, expressed by the supply chain relationships (SCR) (H5b: γ = 0.284; t = 6.428; p<0.001). The second factor expressing heterogeneity (i.e., value network relationships) was not important for achieving benefits from interfirm collaboration, nor was the originality and formalization of the relational strategy.

We were not able to test Hypothesis 6 because the respondents did not indicate negative outcomes from collaboration, so it was impossible to determine this variable.

## 5. Discussion and conclusion

The results confirm the linkage between the strategic choices, the attributes of a relational strategy, and the benefits of collaboration. In the opinion of the respondents, the relational rent was mainly the market type and yielded benefits resulting from access to new markets and contractors and a stronger market position and more bargaining power with entities outside the relationship, as well as image benefits [110, 117]. Obtaining this rent was conditioned by only two attributes of the relational strategy, i.e., durability and heterogeneity in the supply chain. Thereby, these two attributes are mediators in the relationship between particular strategic choices and achieved benefits from collaboration.

The durability of the relational strategy depends on the commitment of the individual entities in the relationship [58], mutual trust [55] and the benefits obtained [108], which is conducive to long-term relationships. It is therefore a result of value creation according to the logic of the value network, according to which value is created jointly and each partner derives specific benefits from it (relational rent) [109]. It turns out, however, that in the opinion of Polish managers, the durability of the relational strategy is also influenced by value creation according to the value chain, based on vertical interdependence and transactional relations [62]. With this type of relationship, the company tries to maximize its own benefits, especially in the economic dimension (“Burt’s rent”) [144] which should somewhat weaken the durability of these relationships. We find two main reasons for this. First, the transactional relationships showed potential for development and, as a result of being repeated, turned into long-term relationships in which commitment and trust have a calculated dimension. Second, Polish companies still relatively rarely establish coopetitive relationships, which are characteristic of the value network, and thus choose the known chain interdependence, based on which they aim to develop lasting inter-organizational relationships. Regardless of how the value is generated, Polish managers agree that the durability of a relational strategy is ensured by resource-related mechanisms to maximize it [17], causing the increase in economic rent.

The heterogeneity of the relational strategy conditions the achievement of market benefits coming from collaboration only in terms of the heterogeneity of supply chain relationships. According to Polish managers, these benefits are achieved primarily through various relationships with suppliers and customers, rather than in a broader perspective, within a value network, with the criteria for selecting partners being irrelevant here. This is quite surprising, as many studies have indicated that it is the value network and relationships with various external stakeholders (with competitors and complementors in addition to suppliers and customers) that are the source of the relational rent (e.g. [44, 145]). In our opinion, this is a consequence of how Polish companies choose to create value. Value created according to the logic of the value chain affects the durability of the relational strategy, which means that these relationships are not just one-off transactions. Companies focus on achieving the most benefits out of them, including market benefits. In entities where value is created according to the logic of the value network, managers recognize that the heterogeneity of the relational strategy is a result of developing relationships not only with suppliers and customers, but also with other partners who are both within and outside of the economic path, including indirect relationships. Nevertheless, the benefits of collaboration continue to be primarily attributed to relationships with suppliers and customers, i.e., partners with whom they do not directly compete. On the one hand, this may be the result of low relational awareness [8] among Polish managers, while on the other hand, it could be due to the inability to use the potential of the value network [9].

Overall, this study endeavors to make three contributions to the literature on strategic management. First, it tests the perspective of strategic choices by the relational context, indicating that key strategic choice which is decisive for a company to obtain a relational rent is the choice of how to create and appropriate value. Second, this choice directly affects the durability of the relational strategy and—indirectly—its heterogeneity, which are decisive for obtaining a relational rent. Third, the outcomes of collaboration are seen primarily through the prism of market benefits achieved through the durability of the relational strategy and heterogeneity in the supply chain.

From a practical perspective, the paper supplies guidelines to managers regarding the strategic choices that should be made so that the relational strategy contributes to the company’s relational rent. Creating value, in accordance with the chain logic or the value network logic, and its appropriation by maximizing mechanisms, combined with the selection of different entities (not only key ones) from suppliers and customers results in access to new markets and contractors, a stronger market position and bargaining power, and an improved company image. However, the choice of how to form the strategy is of little importance in whether it is achieved.

Our research is not free from limitations, which set further directions of research. Firstly, the research was conducted on Polish companies, so the conclusions can only be read in relation to them. It would therefore be justified to carry out similar research in other cultural contexts, in more and less developed countries, in order to compare the relationships between strategic choices, the attributes of a relational strategy, and the outcomes achieved. This comparison would indicate which of the dependencies have a universal dimension and which of them depend on the cultural context. In particular, a cross-cultural comparison should be interesting, especially that in literature such problems are actual discussed [146, 147].

Secondly, due to the fact that the benefits achieved through collaboration, as our research has shown, are primarily the result of durable and heterogeneous relationships with suppliers and customers, without taking into account the broader potential of the value network, an interesting direction for further research would be to test individual relationships among managers with different levels of relational awareness. Moreover, our research focused only on external stakeholders, however, internal ones, including employees and managerial staff are also involved in strategic processes [148–150]. Therefore, future research including this issue should be also an attractive research direction.

Thirdly, the limitations of the adopted research method (e.g., subjectivity in the respondents’ statements, research conducted at a specific point in time) suggest that it would be valuable to undertake studies using other methods—e.g., longitudinal case studies—which would allow for a deeper explanation of the identified linkage taking into account the duration of individual relationships. These directions for further research do not exhaust all possibilities, and the relational strategy will remain an interesting subject for academic exploration in the future.

## Supporting information

S1 Appendix Factor loadings, convergent validity, and reliability of latent variables. (DOCX)
