# Supplementary material for: A company’s relational strategy: Linkage between strategic choices, attributes, and outcomes
Source: PLoS One. 2021 Jul 22;16(7):e0254531. doi: 10.1371/journal.pone.0254531 (PMC8297868; doi:10.1371/journal.pone.0254531)
Supplement: S1 Appendix — (DOCX) [file pone.0254531.s001.docx]

**S1 APPENDIX**

**Factor loadings, convergent validity, and reliability of latent variables**

| **Variable / Items** | | Loading | Convergent validity | Reliability |
| --- | --- | --- | --- | --- |
| *Value creation by value chain logic (VCC)* | | | | |
| VCC1 | In our company, a value created primarily on the basis of vertical interdependence (i.e. on the supplier-customer line), brings us financial benefits | 0.945 | AVE = 0.771 | α = 0.905  CR = 0.909 |
| VCC2 | In our company, a value created primarily on the basis of vertical interdependence (i.e. on the supplier-customer line), brings us non-financial benefits | 0.928 |  |  |
| VCC3 | We strive to maximize our own benefits in each individual transaction | 0.748 |  |  |
| *Value creation by value network logic (VCN)* | | | | |
| VCN1 | In our company, value created jointly, through collaboration with selected (key for us) suppliers, customers, competitors and other market entities brings financial benefits to each of us | 0.934 | AVE = 0.861 | α = 0.949  CR = 0.949 |
| VCN2 | In our company, value created jointly, through collaboration with selected (key for us) suppliers, customers, competitors and other market entities brings non-financial benefits to each of us | 0.922 |  |  |
| VCN3 | We strive to maximize the mutual benefits in each individual transaction | 0.928 |  |  |
| *Value Appropriation by value protection (VAP)* | | | | |
| VAP1 | We protect part of the value created jointly with our partners through formal contracts | 0.662 | AVE = 0.779 | α = 0.929  CR = 0.932 |
| VAP2 | We protect part of the value created jointly with our partners through hidden knowledge | 0.882 |  |  |
| VAP3 | We protect part of the value created jointly with our partners through secrecy | 0.990 |  |  |
| VAP4 | We protect part of the value created jointly with our partners through confidentiality | 0.960 |  |  |
| *Value appropriation by value maximization (VAM)* | | |  |  |
| VAM1 | We maximize value by having unique (exceptional) resources | 0.639 | AVE = 0.693 | α = 0.852  CR = 0.868 |
| VAM2 | We maximize value by having resources that complement partners' resources | 0.977 |  |  |
| VAM3 | We maximize value by having substitution (similar) resources | 0.847 |  |  |
| *Partner Type (PT)* | | |  |  |
| PT1 | We constantly maintain relationships with various entities | 0.839 | AVE = 0.688 | α = 0.861  CR = 0.868 |
| PT2 | We are actively developing cooperative relationships with key suppliers | 0.782 |  |  |
| PT3 | We are actively developing cooperative relationships with key customers | 0.865 |  |  |
| *Criteria of partners' selection (CPS)* | | | | |
| CPS1 | We select partners for cooperation due to the convergence of cooperation goals (strategic alignment ) | 0.990 | AVE = 0.739 | α = 0.930  CR = 0.930 |
| CPS2 | We select partners for cooperation due to the resources (resource alignment) | 0.997 |  |  |
| CPS3 | We select partners for cooperation due to the extent of trust in them | 0.954 |  |  |
| CPS4 | We select partners for cooperation due to the entity's reputation/prestige | 0.490 |  |  |
| CPS5 | We select partners for cooperation due to the strong position of the entity on the market | 0.754 |  |  |
| *Transactional cooperation (TC)* | | | | |
| TC1 | Our cooperation with suppliers is primarily transactional (based on price) | 0.637 | AVE = 0.552 | α = 0.744  CR = 0.777 |
| TC2 | Our cooperation with customers is primarily transactional (based on price) | 0.963 |  |  |
| TC3 | Our cooperation with other non-competitive partners is primarily transactional (based on price) | 0.567 |  |  |
| *Partnership cooperation (PC)* | | | | |
| PC1 | Our cooperation with suppliers is primarily of a partnership nature (based on long-term cooperation) | 0.652 | AVE = 0.567 | α = 0.779  CR = 0.778 |
| PC2 | Our cooperation with customers is primarily of a partnership nature (based on long-term cooperation) | 0.972 |  |  |
| PC3 | Our cooperation with non-competitive partners is primarily of a partnership nature (based on long-term cooperation) | 0.576 |  |  |
| *Coopetition (Coo)* | | | | |
| Coo1 | If competitors propose cooperation, we accept it (if it is legal and beneficial) | 0.732 | AVE = 0.524 | α = 0.752  CR = 0.767 |
| Coo2 | We cooperate with competitors on our initiative | 0.706 |  |  |
| Coo3 | We cooperate with competitors on customers' demand | 0.733 |  |  |
| *Deliberate way of relational strategy creating (DW)* | | | | |
| DW1 | In our company, activities related to the creation of the relational strategy are planned | 0.915 | AVE = 0.685 | α = 0.788  CR = 0.811  r=0.668 |
| DW2 | In our company, activities related to the creation of the relational strategy are cyclical | 0.730 |  |  |
| *Emergent way of relational strategy creating (EW)* | | | | |
| EW1 | In our company, activities related to the creation of the relational strategy are spontaneous (during company operations) | 0.898 | AVE =0.801 | α = 0.894  CR = 0.889  r= 0.889 |
| EW2 | In our company, activities related to the creation of the relational strategy are ad hoc (during sensing and seizing opportunities) | 0.892 |  |  |
| *Durability (D)* | | | | |
| D1 | Our strategy includes only long-term relationships | 0.842 | AVE = 0.788 | α = 0.930  CR = 0.930 |
| D2 | Our strategy includes relationships in which trust in the partner is high | 0.923 |  |  |
| D3 | Our strategy includes relationships in which the partner shows commitment to cooperation | 0.909 |  |  |
| D4 | Our strategy includes relationships we benefit from | 0.875 |  |  |
| *Heterogeneity: Supply Chain Relationships (SCR)* | | | | |
| SCR1 | Our strategy includes relationships with many entities (not only the key ones for us) | 0.945 | AVE = 0.799 | α = 0.911  CR = 0.922 |
| SCR2 | Our strategy includes relationships with suppliers | 0.740 |  |  |
| SCR3 | Our strategy includes relationships with customers | 0.979 |  |  |
| *Heterogeneity: Value Network Relationships (VNR)* | | | | |
| VNR1 | Our strategy includes relationships with various types of partners who we cooperate with | 0.845 | AVE = 0.652 | α = 0.835  CR = 0.846 |
| VNR2 | Our strategy includes various forms of relationships (e.g. consortia, clusters, franchise, outsourcing, and others) | 0.905 |  |  |
| VRN3 | Our strategy includes indirect relationships, i.e. partners of our partners | 6.651 |  |  |
| *Originality (O)* | | | | |
| O1 | Our strategy is based on the selection of unique/valuable partners | 0.902 | AVE = 0.623 | α = 0.804  CR = 0.763  r= 0.713 |
| O2 | Our strategy is based on forms and principles of collaboration that are difficult to imitate | 0.657 |  |  |
| *Positive outcomes (PO)* | | | | |
| PO1 | Our strategy results in access to new markets/new customers | 0.779 | AVE = 0.519 | α = 0.717  CR = 0.715 |
| PO2 | Our strategy results in access to new contractors (new partners) | 0.728 |  |  |
| PO3 | Our strategy results in improving the firm's image | 0.707 |  |  |
| PO4 | Our strategy results in strengthening market position | 0.705 |  |  |
| PO5 | Our strategy results in raising funds (joint investments) | 0.682 |  |  |
| PO6 | Our strategy results in increasing bargaining power in relation to entities outside the relationships’ layout | 0.719 |  |  |
| PO7 | Our strategy results in a sense of greater security in a competitive situation | 0.711 |  |  |

AVE: Average variance extracted; α: Cronbach's alpha; CR: Composite reliability; r: correlation
